# Semantic segmentation using synthetic images of underwater marine-growth

**DOI:** 10.3389/frobt.2024.1459570

**Published:** 2025-01-08

**Authors:** Christian Mai, Jesper Liniger, Simon Pedersen

**Affiliations:** AAU Energy, Aalborg University, Esbjerg, Denmark

**Keywords:** unmanned underwater vehicles (UUV), synthetic images augmentation, semantic segmentation, virtual environment, underwater operations, marine growth, fouling

## Abstract

**Introduction:**

Subsea applications recently received increasing attention due to the global expansion of offshore energy, seabed infrastructure, and maritime activities; complex inspection, maintenance, and repair tasks in this domain are regularly solved with pilot-controlled, tethered remote-operated vehicles to reduce the use of human divers. However, collecting and precisely labeling submerged data is challenging due to uncontrollable and harsh environmental factors. As an alternative, synthetic environments offer cost-effective, controlled alternatives to real-world operations, with access to detailed ground-truth data. This study investigates the potential of synthetic underwater environments to offer cost-effective, controlled alternatives to real-world operations, by rendering detailed labeled datasets and their application to machine-learning.

**Methods:**

Two synthetic datasets with over 1000 rendered images each were used to train DeepLabV3+ neural networks with an Xception backbone. The dataset includes environmental classes like seawater and seafloor, offshore structures components, ship hulls, and several marine growth classes. The machine-learning models were trained using transfer learning and data augmentation techniques.

**Results:**

Testing showed high accuracy in segmenting synthetic images. In contrast, testing on real-world imagery yielded promising results for two out of three of the studied cases, though challenges in distinguishing some classes persist.

**Discussion:**

This study demonstrates the efficiency of synthetic environments for training subsea machine learning models but also highlights some important limitations in certain cases. Improvements can be pursued by introducing layered species into synthetic environments and improving real-world optical information quality—better color representation, reduced compression artifacts, and minimized motion blur—are key focus areas. Future work involves more extensive validation with expert-labeled datasets to validate and enhance real-world application accuracy.

## 1 Introduction

Semantic segmentation is the task within machine vision used to analyze the extent of objects in a given scene on a pixel level. It is widely applied within various robotics contexts (Juana Valeria Hurtado and Abhinav Valada, 2024). For example, it has applications within the inspection of surfaces in industrial applications (Joshi et al., 2022) and landcover identification in remote sensing (Medellin et al., 2023; Paul et al., 2012) gathered from unmanned aerial vehicles (UAV’s), or for the identification of safety-critical object information, such as in autonomous agricultural field robotics (Mujkic et al., 2023).

In summarizing, no matter the domain, semantic segmentation is used to find the extent of complex objects within a scene; in modern applications, the segmentation task is often implemented using Deep-Learning strategies, especially within robotics, a review of which is provided in Ni et al. (2023). In supervised deep-learning segmentation strategies, a neural network is trained on labeled datasets (subject to the availability of sufficient computational power for training and execution) (Juana Valeria Hurtado and Abhinav Valada, 2024). Underlying the image segmentation networks is often an image classification neural-network architecture, such as the one developed for the identification of marine-fouling species in Chin et al. (2017). In the underwater domain, it has applications within structural inspection (O’Byrne et al., 2015), which is important to mitigate potential structural failures from, e.g., cracks (O’Byrne et al., 2018a), and within biology such as remote visual species identification (LutzKrause et al., 2023) (similar to the terrestrial remote sensing case); here data is often gathered using unmanned underwater vehicles (UUV’s), specifically either tethered remote operated vehicles (ROV’s) or autonomous underwater vehicles (AUV’s) (Pedersen et al., 2022). A systematic review of underwater object detection using deep-learning methods is provided in Xu et al. (2023), an added exploration of the relation between pre-processing image enhancement and the subsequent performance of deep-learning methods, showing that there is not always a positive correlation.

In submerged environments, obtaining controlled experimental labeled survey data is challenging, partly due to the substantial cost involved and partly due to the many uncontrolled environmental influences, such as turbidity, signal attenuation, and variable lighting, which are also driven by uncontrollable weather effects (O’Byrne et al., 2018b; O’Byrne et al., 2018a). This is particularly troublesome in coastal and near-surface work situations (Liniger et al., 2022). Noteworthy real-world labeled benchmark datasets for general underwater segmentation include SUIM (Islam et al., 2020), and for ship-hull inspection, LIACi (Waszak et al., 2023). Training of segmentation algorithms on the SUIM dataset for both fully supervised and semi-supervised training has been explored in several works (Kumar et al., 2024), explored the use of unsupervised pseudo-segmentation as a downstream task and compared the performance against state-of-art unsupervised dense segmentation, finding an improvement of ≈ 15% when measured on mean-intersection-over-union on the SUIM dataset (Vorndran and Roeck, 2024). explored the use of inconsistency masks (IM) to improve the segmentation mask performance in areas where the models have difficulty in segmentation, such as around the edges of objects, and found a possibility to approach the full data-set training performance using only a limited subset. Xu et al. (2024) explore the use and adaptation of Segment Anything Machine (SAM) to create AquaSAM, a model tuned for foreground/background segmentation which yielded an improvement of ≈ 7% *versus* baseline SAM-ViTB. The related task of multi-label classification, based on the LIACi dataset, was explored in (Azad et al., 2023), but outside this has not yet been explored extensively in the literature.

In many different application contexts, both industrial, commercial, and defense settings, where similar constraints on the gathering of data exist, the utilization of synthetic environments for machine-learning training has proven to be fortuitous (Sergey, 2021; O’Byrne et al., 2018b). Synthetic data for training entails a cost reduction compared to real-world submerged operations, precise control over the environment, and the ability to simultaneously generate accurate ground truth without using any human resources for labeling. Graphical modeling and rendering tools, focusing primarily on visual rendering for various artistic and commercial purposes, hold importance for achieving photorealistic rendering, a crucial aspect of machine learning tasks. Examples include VUE, utilized in studies like (O’Byrne et al., 2018b), and Blender, featured in research such as (Smith et al., 2022). These tools contribute significantly to creating visually realistic submerged environments, essential for machine learning applications (Sergey, 2021).

A typical challenge in submerged environments is marine growth fouling, which is problematic in many offshore contexts, including causing increased mechanical weight and wave load on offshore structures such as foundations, structural jackets, and mooring lines (Pedersen et al., 2022). Additionally, marine growth can hinder non-destructive inspection processes in such domains by occlusion of structural surfaces (Liniger et al., 2022; O’Byrne et al., 2018a). In a related scenario, marine fouling can also cause additional drag on ship hulls as noted in (Waszak et al., 2023; International Maritime Organization IMO, 2010). To meet operational demands, management of marine growth is often necessary, including derivation of the species and coverage (Pedersen et al., 2022; Liniger et al., 2022). This task can be conducted autonomously using UUVs with onboard processing, thus alleviating the need for high-cost high-risk human diving operations previously used (Liniger et al., 2022; João Afonso Borges Carvalho, 2023).

Based on this problem of marine fouling management, this work explores the use of visual, 2D semantic segmentation algorithms to segment images of marine growth on various scenarios, thereby enabling the automatic determination of the species coverage in scenes while avoiding manual human labeling and video and image analysis, thus facilitating various control measures, such as calculations of structural loads or cleaning intervals. In line with the objective to alleviate the need for labor-intensive manual pixel-level labeling of training materials, a purely synthetic-image approach is used for training the segmentation network. In contrast to the work of O’Byrne et al. (2018b), no adjunctive machine learning is used to refine the results of the neural network; however real-world images are used in the initial classifier training, rather than relying purely on synthetic data for all training steps. Unlike the approach of (LutzKrause et al., 2023; Lin et al., 2022; Alonso et al., 2019), no manual or semi-automatic labeling of the segmentation mask was performed on real-world imagery, with the segmentation mask instead being purely automatically defined in synthetic data, thus completely automating the segmentation dataset labeling process.The main contributions of this study are:

• Training and quantitative evaluation of the performance of two underwater segmentation networks on synthetic segmentation data.

• Evaluation of synthetic-only segmentation against expert-labeled ground-truth datasets: LIACi (Waszak et al., 2023) and SUIM (Islam et al., 2020) and their accompanying network performances.

• A qualitative evaluation of the real-world performance of these networks on images from three distinct underwater scenarios using our own field data:
  – Inspection of an offshore structure (Liniger et al., 2022).
  – Inspection of harbor surfaces (wall and mooring post).
  – Inspection of ship hulls (Waszak et al., 2023).

Extending the case studies of Mai et al. (2024), this work deepens the methodological description of the synthetic image training approach for classification underwater and provides additional validation by the following additions:

• Evaluation of a commissioned synthetic dataset of ship hulls with varied structural paint textures

• New species of attached marine growth representing invasive ship hull fouling

The remainder of the paper is organized as follows: Firstly, the materials and methods of the work are described, which includes the synthetic dataset used, the real-world validation data, the neural-network architecture and training; secondly, the results of the trained neural network segmentation are illustrated and evaluated 1) on the synthetic dataset itself, 2) on the expert-labeled datasets, and 3) applied to our data from the real-world environments in selected Danish harbors and offshore sites, where marine fouling is present; finally, some concluding remarks are given as well as perspectives for network implementation and future applications.

## 2 Materials and methods

Synthetic datasets are used to perform transfer learning on a pre-trained neural network architecture and subsequently evaluated on the real-world out-of-bag test data. Firstly, the classes to be included in the segmentation task must be considered, as well as the required resolution of the input and output stages of the neural network based on the available data.

### 2.1 Classes and species considered

Based on the environment of interest being a submerged structural inspection task, the classes of interest can be delineated between the background environment, the structural components, and the attached marine growth. The marine-growth classes are selected based on the prevalent fouling species in the Danish North Sea. A distinction can be made between hard and soft marine growth, with hard marine growth characterized as incompressible, and soft marine growth as easily compressible, since they have different structural load characteristics as described in Gaurier et al. (2014).

Based on these considerations, the following classes have been applied to the segmentation task, corresponding to the underlying datasets’ classes. The offshore dataset and underlying rendering methodology for both datasets are described in Mai et al. (2024), which consists of the creation of 3D scenes of the respective environment in Blender, with the inclusion of accurate water, structural, and marine-growth 3D models. For the ship-hull dataset, from (Atlantic Tech and Candy GmBH, 2023), the sea squirts were included as an additional species, due to their presence in the reference video materials. An overview of the classes used in the segmentation training and validation datasets is given in Table 1.

**TABLE 1:** Segmentation class descriptions, 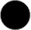 denotes separately labeled sub-category, 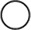 denotes absence, 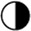 denotes present but labeled by major category.

Category	Subcategory	Description/species	Offshore dataset (Mai et al., 2024)	Ship dataset (Atlantic Tech and Candy GmBH, 2023)	LIACi (Waszak et al., 2023)	SUIM (Islam et al., 2020)
Hard marine growth	Blue mussels	*Mytilus edulis*	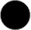	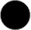	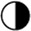	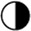
	Balanus	*Balanus balanus*	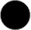	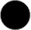	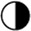	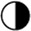
Soft marine growth	Seagrass	*Zosteraceae*	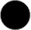	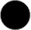	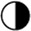	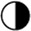
	Seaweed/macroalgae	*Phaeophyceae*	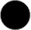	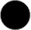	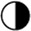	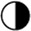
	Sea anemones	*Actiniaria*	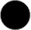	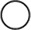	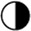	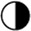
	Sea squirts	*Ascidiacea*	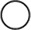	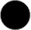	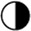	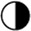
Seawater	Background	Unobstructed water-volume	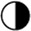	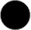	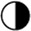	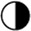
	Seafloor	Seafloor, nominally sand	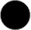	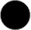	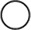	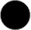
	Water surface	Unobscured water surface	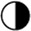	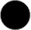	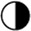	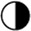
	Marine snow	Particulates in the water	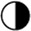	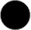	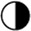	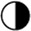
Structure	Shiphulls, structures, and wrecks	Man-made structure	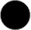	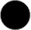	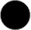	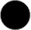

**FIGURE 1:**
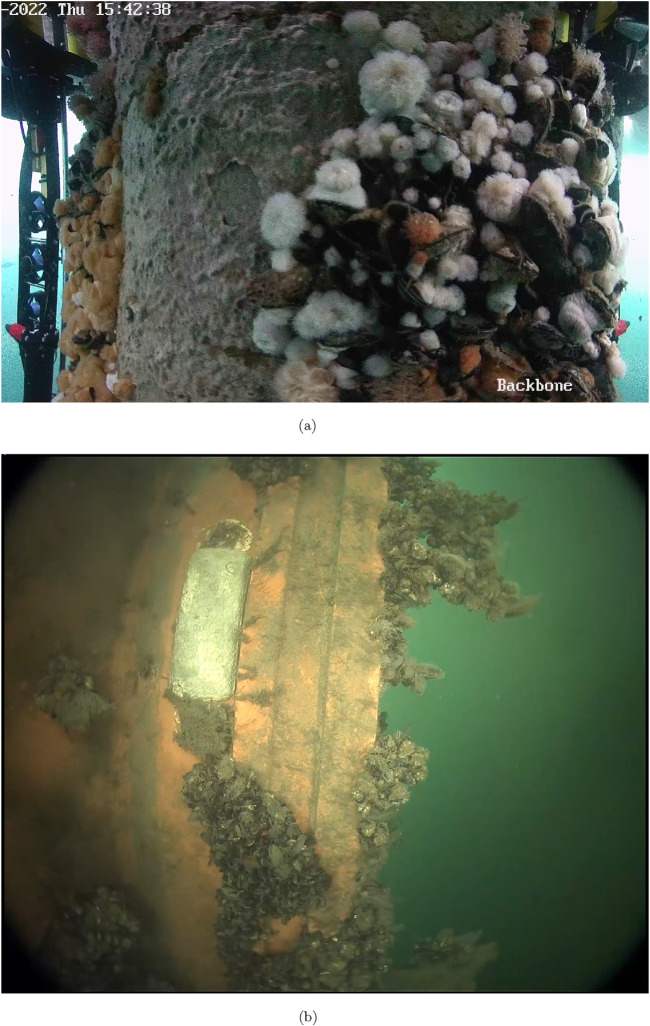
Real-world marine-growth examples. **(A)** Example of offshore structural marine fouling (courtesy of SubC Partners). **(B)** Example of ship hull marine fouling (courtesy of Blue Atlas Robotics).

An example of marine fouling on a Danish offshore structure is illustrated on Figure 1A. The left side of the image shows a section of the previously cleaned structure with only a thin layer of algal fouling remaining. In contrast, the right side constitutes heavy fouling with a mix of anemones, mussels, and seaweed. An example of marine fouling on a ship hull is shown in Figure 1B, where the fouling consists of mussels, sea squirts, and balanus.

### 2.2 Synthetic image dataset

We have utilized two synthetic image datasets consisting of > 1000 rendered images each, commissioned from Atlantic Tech and Candy GmBH (2023), which is generally considered sufficient for transfer learning (Shahinfar et al., 2020), to train the segmentation neural networks, which will be evaluated in Section 3. Recalling from Mai et al. (2024), the datasets are based on virtual environments created in, and rendered using, the open-source software Blender, with the Cycles raytracing engine (Blender Documentation Team, 2023); the virtual environments themselves are based on CAD-models and a mixture of 3D scanned and procured models and textures from commercial asset providers. The rendering of images and labels is conducted through procedural scripting, integrated with the rendering process steps, thus pixel-level labels are generated automatically and exactly for each rendered scene, corresponding to the classes given in Section 2.1. In the datasets, the rendering distances and water types have been varied to cover various inspection scenarios encountered in real-world video inspections, based on the reference materials gathered using ROVs provided by the industrial partners. This robustifies the trained algorithm so it does not overfit towards the appearance of the marine species and structural surfaces at a single, given distance. To include the variations in turbidity and water coloration in different real-world operating scenarios, varied Jerlov water types have been used during rendering, from very clear Jerlov I oceanic water to coastal Jerlov 5C water, see (Mai et al., 2024) for example, images. For additional augmentation, both noisy and denoised (using NVIDIA OptiX denoiser) images have been used, as well as images with no water volume. In addition, for the second dataset (ship hulls), the focal length has been varied for each image. A detailed description of the synthetic dataset creation can be found in Mai et al. (2024), and the main characteristics are recalled below:

• Image quantity: 1038 × 2

• Image size: 532 × 299pixel

• Range to target surface: 30100 cm

• Data augmentation: noisy and denoised images

• Water types (Jerlov): I, II, III, 1C, 3C, 5C

• Included classes: See Section 2.1.

### 2.3 Neural-network architecture

The deep-neural network is re-applied from the previous case demonstration of (Mai et al., 2024); the deep-neural network chosen for the segmentation task is DeepLabV3+ segmentation network (details in (Chen et al., 2018)) with an Xception classifier backbone (details in (Chollet, 2017)), where the classification backbone has been trained on real-world marine-fouling images. The choice of network architecture is guided by the current state-of-art, as described in (Sergey, 2021), where several applications of synthetic data and various network architectures have been evaluated, including DeepLabV3+, and the strengths and weaknesses of the networks are illustrated. Additionally, DeepLabV3 is widely applied in similar underwater segmentation works (Waszak et al., 2023; Islam et al., 2020), thus readily allows for comparison of the synthetic-only transfer-learned network performance. The neural network architecture has been modified by the replacement of the pixel classification layer to compensate for the class imbalance in the synthetic datasets; the class imbalance and resulting weights are illustrated in Tables 2, 3 for the offshore and ship hull case respectively.

**TABLE 2:** Pixel wise class imbalance and layer weights - Offshore case.

Name	Pixel Count	Image Pixel Count	Image Freq	Class Weights
seawater	5.9642e+06	3. 2068e+08	0.018599	7.8879
seafloor	1.4286e+06	1. 1994e+08	0.011911	12.316
structure	1.0403e+08	3.2068e+08	0.3244	0.45223
mussels	3.2484e+07	2.2142e+08	0.14671	1
balanus	1.2531e+06	1. 9279e+08	0.0064998	22.571
seaweed	1.1736e+08	3.2036e+08	0.36634	0.40047
anemones	5.8161e+07	2.0997e+08	0.277	0.52963

**TABLE 3:** Pixel wise class imbalance and layer weights - Ship case.

Name	Pixel Count	Image Pixel Count	Image Freq	Class Weights
Seawater	9.4391e+07	5.12e+08	0.18436	0.76053
seafloor	2.828e+07	3.0976e+08	0.091298	1.5357
structure	1. 6781e+08	5.12e+08	0.32775	0.4278
mussels	6.1969e+07	5.12e+08	0.12103	1. 1585
balanus	8.5624e+06	5.1149e+08	0.01674	8.3757
seaweed	7.9417e+07	5.1149e+08	0.15527	0.90303
sea_squirts	7.1572e+07	5.1046e+08	0.14021	1

### 2.4 Deep-neural network training

**FIGURE 2:**
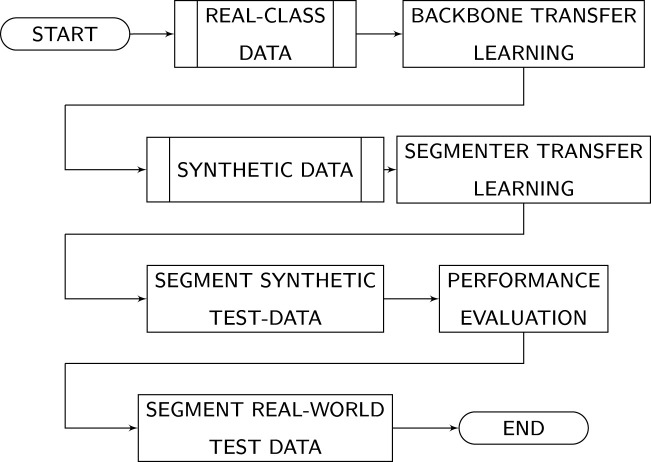
Training and test process, adapted from (Mai et al., 2024).

An overview of the methodology is presented on Figure 2, which illustrates the sequential nature of the pre-training, transfer learning, and the subsequent evaluation of synthetic and out-of-bag real-world data.

#### 2.4.1 Backbone training

Ideally, a pre-trained classification network covering all the classes included in Section 2.1 would be applied as the backbone classifier, but such a network is not readily available. In substitution, an Xception classifier, pre-trained on the ImageNet database, has been additionally transfer-learned on the dataset of (Chin et al., 2017). The xception backbone has been chosen for its best-in-class classification accuracy and the ability to be directly inserted into the DeepLabV3+ segmentation network. Through the transfer learning on the real-world image dataset, the training performance of the overall segmentation network is increased, including a reduction in the time to convergence.

#### 2.4.2 Segmentation training

The segmentation network is DeepLabV3+ with the pre-trained xception backbone inserted (Mai et al., 2024). The dataset has been split utilizing 60% as training images, with 20% validation and 20% testing images set aside. The images have been augmented using randomized rotation in the range −180180deg, and both denoised and noise images have been included as additional augmentation. Cross-entropy loss is utilized in the training.; as in Mai et al. (2024), the training is performed with a Stochastic gradient descent with momentum (SGDM) solver, constant learning rate of 0.001, validation frequency of 10, and validation patience of 5. The learning rate selection is informed by the transfer learning scenario, so the learning rate should be set lower than from-scratch learning (Bengio, 2012); similar learning rates have been found through optimization in Cai et al. (2022); Liu et al. (2024); Liu et al. (2021) thus providing a basis for the selection.

**FIGURE 3:**
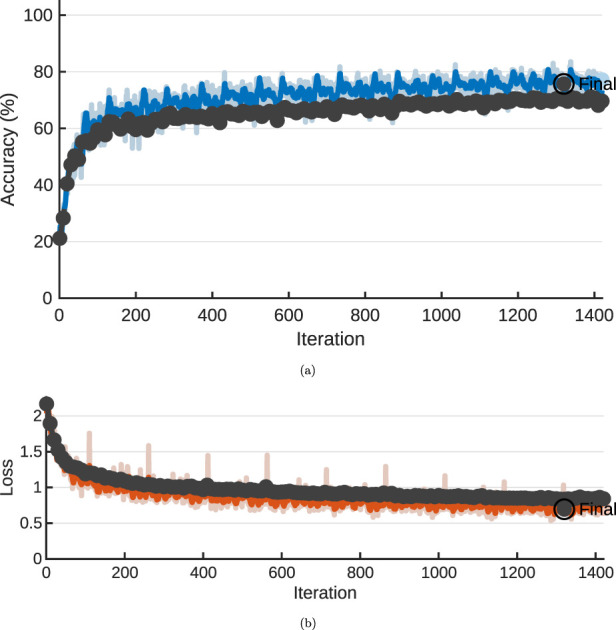
Training progress, offshore case, NVIDIA T4, 10 epochs maximum. **(A)** Training accuracy. **(B)** Training loss.

**FIGURE 4:**
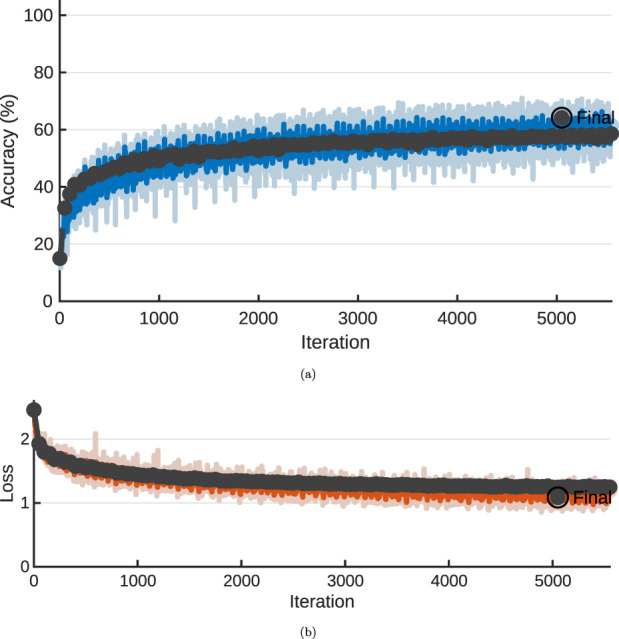
Training progress, ship-hull case, NVIDIA RTX4090, 100 epochs maximum. **(A)** Training accuracy. **(B)** Training loss.

The training was performed on a single NVIDIA GPU using MATLAB GPU accelerated training, and the training graph from training the network is shown on Figures 3, 4 for the two datasets respectively. In figure Figures 3A, 4A, the training accuracies is illustrated, and on Figures 3B, 4B, the losses, respectively. The total training was completed in 1420 iterations for the offshore case and 5210 iterations for the ship hull case. The training was terminated due to meeting the validation requirements in both cases, to avoid overfitting. Training for both datasets was performed in a similar time, varying between 141148min or ≈ 2 h 20min.

### 2.5 Test sites: Offshore, coastal and ship-hulls

**FIGURE 5:**
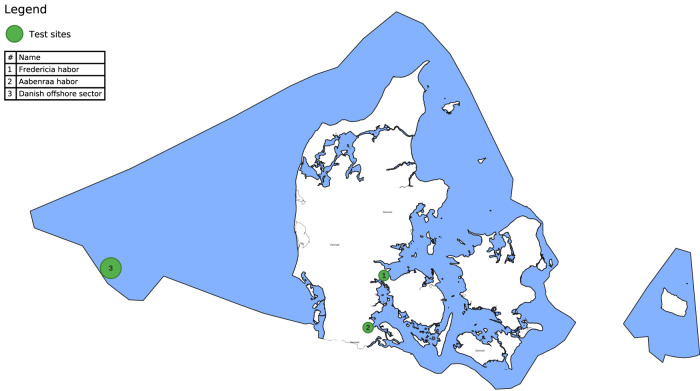
Overview of test site locations, 1 and 2 are coastal sites (harbors), and 3 is an offshore site (location approximate).

For qualitative assessment of the developed algorithms, data from three test sites has been utilized: one environment near an offshore installation in the Danish North Sea and two harbor locations, Aabenraa and Fredericia, on the east coast of Denmark, as marked on the map of Figure 5. These sites serve as typical operating cases where marine growth is commonly found, i.e., on long-term fixed structures such as harbor walls and caissons, and where a representative species distribution of marine growth is expected to be present based on previously gathered video reference materials and the marine growth species description of Gaurier et al. (2014). The primary characteristics of the test sites are listed in Table 4, where the turbidity and depth have been retrieved from satellite sources at (Copernicus Marine Service, 2023) and verified by local measurements. In addition, several videos of ship-hulls from various locations, provided by our industrial partners, have been used for validation.

**TABLE 4:** Test site characteristics.

Site #	Name	Location	Maximum depth	Turbity range	Expected type (Jerlov)
1	Coastal - Fredericia Habor	55.5578494N, 9.7337925E	11m	<1.5 FTU	3C–5C
2	Coastal - Aabenraa Habor	55.0254519N, 9.4409781E	18m	<0.9 FTU	3C–5C
3	Offshore - North Sea	≈ 56N, ≈ 4.5E	40m	N/A	II

## 3 Results

The segmentation network has been tested on three scenarios: 1) the data of the synthetic test sets (set-aside), 2) images from two expert-labeled datasets, 3) images from real-world out-of-bag scenarios; an offshore installation, a selection of ship hulls, and images from two coastal installations in harbors.

### 3.1 Test results - Synthetic images

The synthetic image test sets for the offshore and ship hull cases have been segmented using the two trained networks.

#### 3.1.1 Offshore case

**FIGURE 6:**
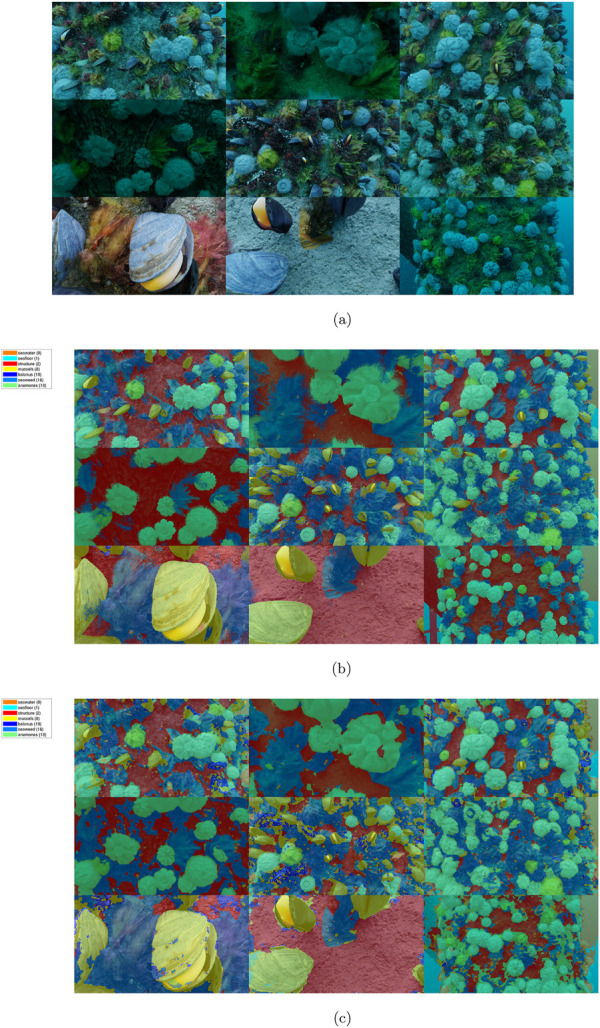
Montage of segmented synthetic images, RGB and ground truth from (Atlantic Tech and Candy GmBH, 2023). **(A)** RGB input image. **(B)** Ground truth mask overlay. **(C)** DeepLabV3+ segmentation.

An example of the segmentation results of the offshore synthetic image test set is shown on Figure 6, providing an overview of the input images, ground truth, and output segmentation for comparison.

**FIGURE 7:**
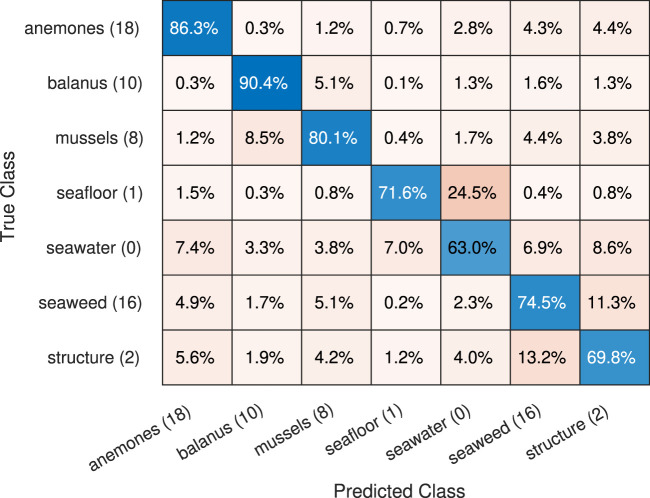
Confusion matrix, offshore structure, updated from (Mai et al., 2024).

The confusion matrix for the synthetic data, shown on Figure 7, illustrates the row-normalized class accuracy for the offshored structure case. The accuracy is high for most of the marine-growth classes, with an average ≈ 74%, and in all cases above the benchmark (67.78%). A notable false classification occurs between seawater and seafloor; this is partly caused by the relatively featureless textures and the obfuscation of the seafloor at longer distances, which makes the classes’ appearance overlap. The second largest incorrect classification occurs between the structural surfaces and the seaweed, most likely due to the downy texture at the edge of the seaweed, combined with the color and texture similarity between structural surfaces and seaweed. The third incorrect classification is between the balanus and mussels, caused by the balanus’s proximity since they are directly on the mussel surfaces.

#### 3.1.2 Ships hulls case

**FIGURE 8:**
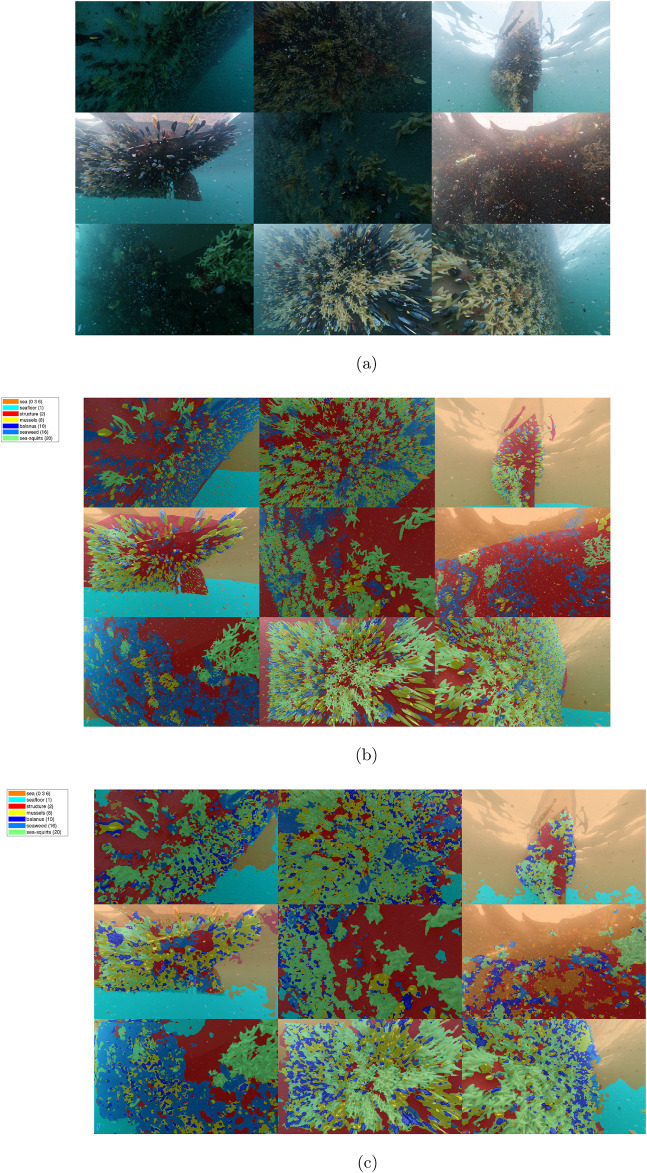
Montage of segmented synthetic images, RGB and ground truth from (Atlantic Tech and Candy GmBH, 2023). **(A)** RGB input image. **(B)** Ground truth mask overlay. **(C)** DeepLabV3+ segmentation.

An example of the segmentation results of the offshore synthetic image test set is shown on Figure 8, providing an overview of the input images, ground truth, and output segmentation for comparison.

**FIGURE 9:**
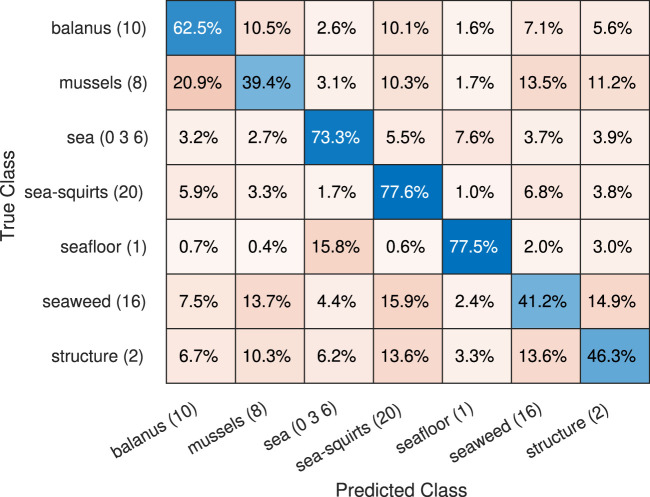
Confusion matrix, ship hulls.

The confusion matrix for the synthetic data, shown on Figure 9, illustrates greatly varying accuracy for the marine-growth classes between 4374%, which is generally lower than the offshore structural case, with some species accuracy below and some above the benchmark (67.78%). The dominant false classification occurs between seawater, seafloor, and marine snow; this is caused by the relatively featureless textures and the obfuscation of the seafloor at longer distances, which makes the classes’ appearance overlap, as well as the randomized location of the marine snow in the images. The second largest incorrect classification is between the balanus and mussels, again caused by the balanus’s proximity since they are directly on the mussel surfaces. A third incorrect classification occurs between the structural surfaces and the seaweed, similar to the offshore case.

### 3.2 Test results - Expert labeled datasets

While datasets that exactly match the applications and segmentation classes synthetic renders of this work are not available, it is possible to perform an alternative quantitative evaluation by using datasets that have overlapping super-classes such as general marine-growth, seawater, and man-made structures; for this purpose, two manually labeled datasets have been retrieved and tested with the developed neural networks for the offshore and ship hull cases respectively, detailed in the sections Section 3.2.1 and Section 3.2.2.

#### 3.2.1 Ship hull labeled dataset

**FIGURE 10:**
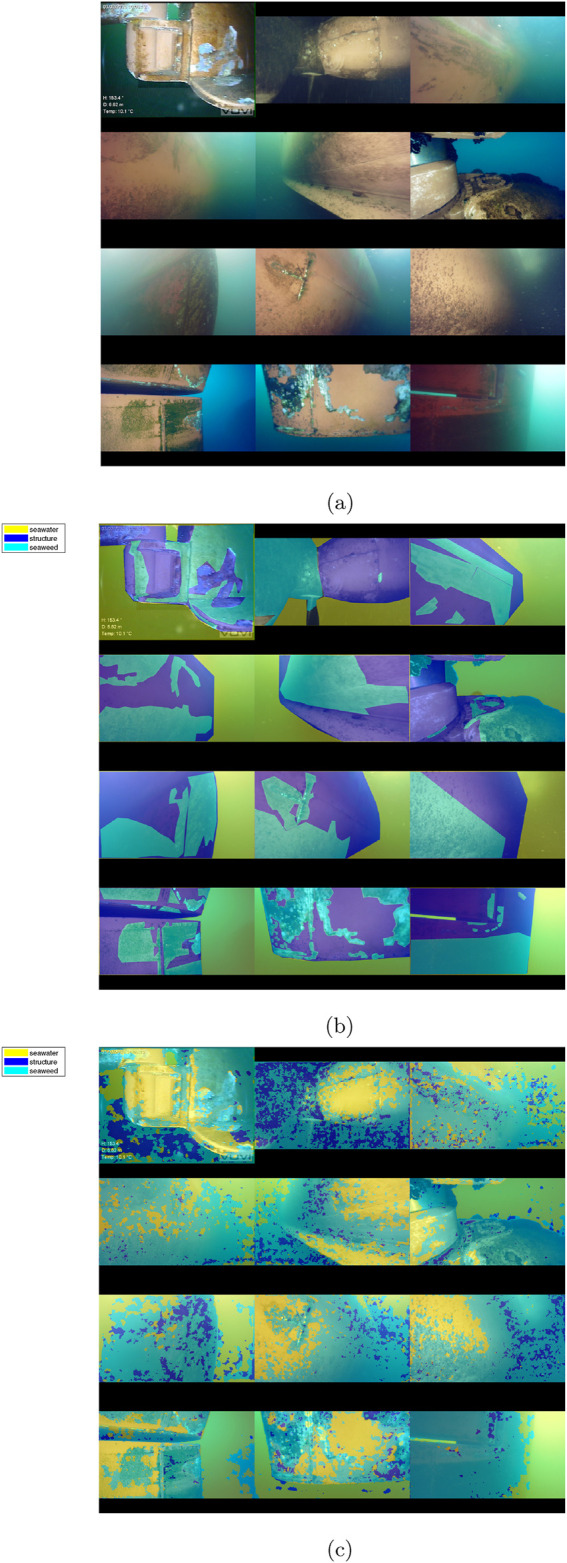
Montage of segmented synthetic images, RGB and ground truth from (Waszak et al., 2023). **(A)** RGB input image. **(B)** Ground truth mask overlay. **(C)** DeepLabV3+ segmentation.

To demonstrate the quantitative performance of the developed neural network for the ship hull case, an expert-labeled dataset will be segmented. A dataset with the exact classes of the synthetic images is not available, so a dataset with the overlapping superclasses of ship-hull, background, and marine growth has been applied as a substitute from the LIACi dataset^1^, available at (Waszak et al., 2023). Since the LIACi dataset does not distinguish marine-growth species, for the purpose of comparison, the output of the neural network has been merged so that marine-growth classes include both the soft and hard marine growth classes (seaweed, mussels, balanus, anemones). To delimit the comparison and presentation, images have been selected from the dataset where at least 20% of the image is the background seawater and at least 20% marine fouling. The output of the segmentation is shown on Figure 10C, with the ground truth on Figures 10B, and RGB input on Figure 10A respectively. In general terms, the performance is good for the detection of the marine-growth classes but it is insufficient for the detection of the structural components, with large misclassification as seawater. Similar to the offshore best cases, the separation of marine growth and seawater background is clearly delimited, but the structural elements are still incorrectly classified as seafloor or seaweed, rather than structure. The accuracy is highest for the true-positive detection of the marine-growth class as well as for the seawater itself, as shown on the row-normalized confusion of Figure 12A, however, the detection of the seafloor and especially structure shows severe misclassification with less than <42.2% <5.3% correctly classified, respectively, rendering the results for these classes unreliable for all presented segmentations. Only the seaweed true-positive rate is above the benchmark case (67.78%). Concerning the segmentation outputs on Figure 10C and comparing with the RGB inputs on Figure 10A, it is evident that the misclassification is most severe when the background seawater is dark (low illumination) and markedly less severe when the background is obscured by low visibility, i.e., high turbidity. The detailed per-class performance metrics for the LIACi dataset are given in Table 5A

**TABLE 5:** (a) Per-class performance metrics for LIACi dataset subset, our network. (b) Per-class performance metrics for SUIM dataset subset, our network. Dataset performance metrics.

	Accuracy	IoU	MeanBFScore
seawater	0.4224	0.2525	0.27564
structure	0.052925	0.044498	0.16937
seaweed	0.74115	0.37775	0.27182

	Accuracy	IoU	MeanBFScore
seawater	0.65622	0.60769	0.48777
seafloor	0.0014823	0.00086848	0.015662
structure	0.021133	0.0042696	0.020863
seaweed	0.75701	0.55868	0.44506

#### 3.2.2 Offshore labeled dataset

**FIGURE 11:**
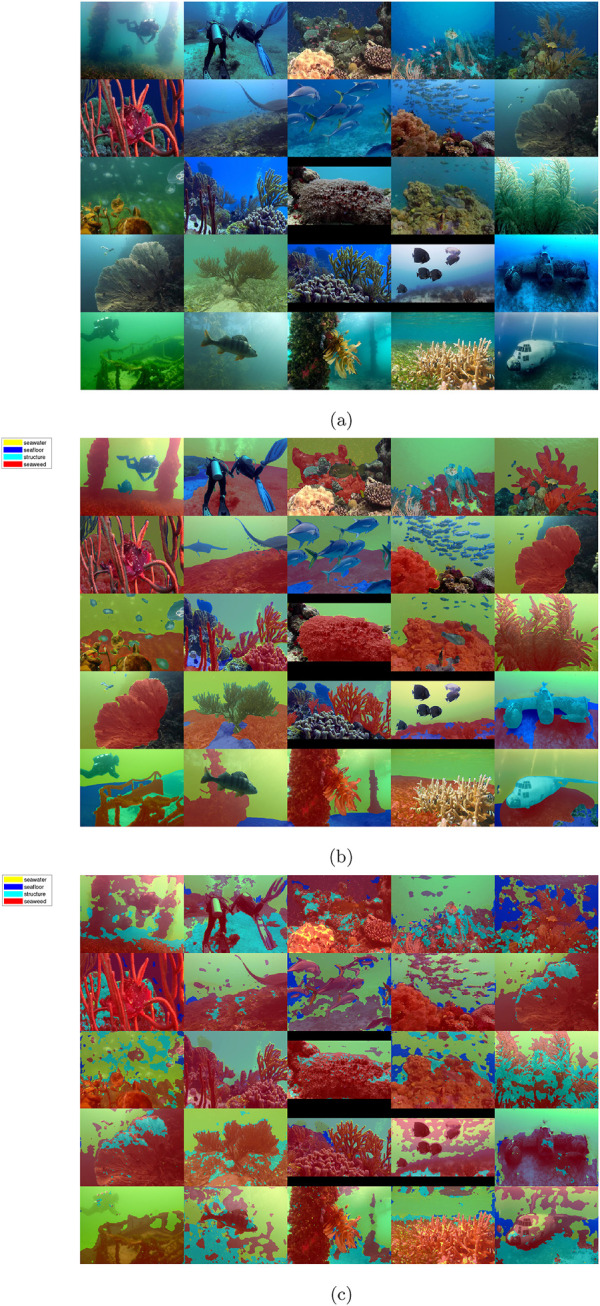
Montage of offshore expert images, RGB and ground truth from (Islam et al., 2020). **(A)** RGB input image. **(B)** Ground truth mask overlay. **(C)** DeepLabV3+ segmentation.

**FIGURE 12:**
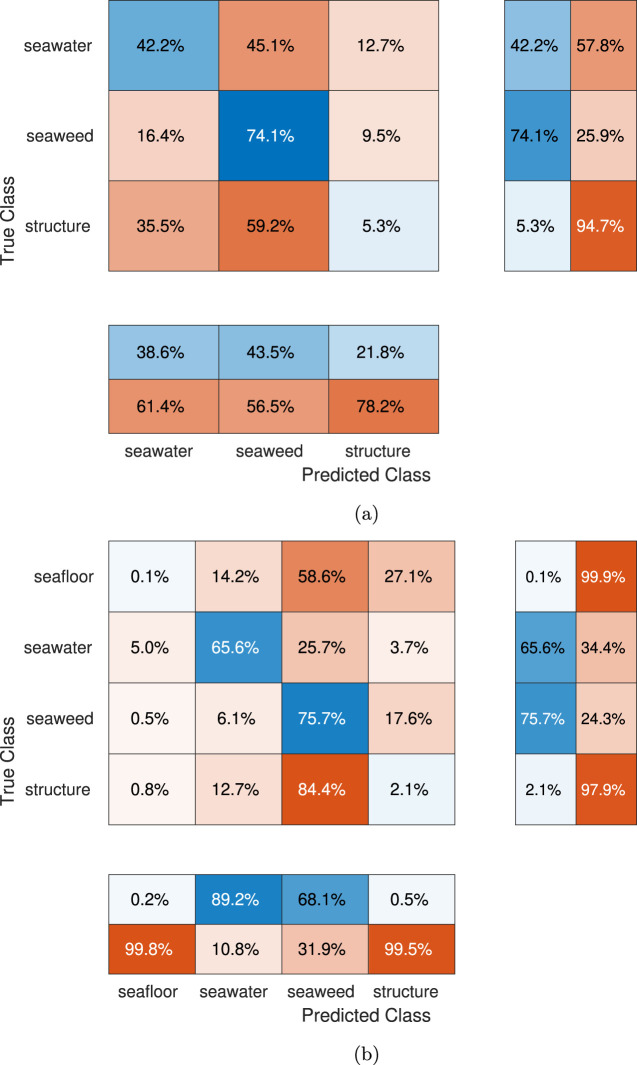
Confusion matrixes for expert labeled dataset subsets. **(A)** Confusion matrix - LIACi dataset, row-normalized. Lower box is classwise precision, right box is class-wise recall. **(B)** Confusion matrix - SUIM dataset, row-normalized. Lower box is classwise precision, right box is class-wise recall.

An expert-labeled dataset will be segmented to quantitatively assess the performance of the developed neural network for the ship hull as a demonstration case. A dataset with the exact classes of the synthetic images is not available, so a dataset with the overlapping superclasses of ship-hull, background, and marine growth has been applied as a substitute from SUIM dataset^2^, available at Islam et al. (2020). Since the SUIM dataset classes do not directly match the trained classes, for the purpose of comparison, the output of the neural network has been merged so that marine-growth classes include both the soft and hard marine growth classes (seaweed, mussels, balanus, anemones), while retaining the classes for seafloor and seawater, and remapping wrecks as structure. To delimit the comparison and presentation, images have been selected from the dataset where at least 20% of the image is the background seawater and at least 20% marine fouling. The output of the segmentation is shown on Figure 11C, with the ground truth on Figures 11B, andRGB input on Figure 11A respectively. In general terms, the performance is good for detecting the marine-growth classes. Still, it is insufficient for the detection of the structural components, with large misclassification as seawater. In the best cases, the separation of seaweed and seawater background is clearly delimited, but the structural elements are still incorrectly classified as seawater rather than structure. The accuracy is high for the true-positive detection of the marine-growth class, as shown on the row-normalized confusion of Figure 12B. However, the detection of seafloor and especially structure shows severe misclassification, rendering the results for these classes unreliable for all presented segmentations. Concerning the segmentation outputs on Figure 11C and comparing with the RGB inputs on Figure 11A, it is, like the ship hull case, evident that the misclassification is most severe when the background seawater is dark (low illumination) and marked, but less severe when the background is obscured by low visibility, i.e., high turbidity. Additional misclassification occurs with the presence of non-trained elements such as human divers and vertebrates. The detailed per-class performance metrics for the SUIM dataset are given in Table 5B.

#### 3.2.3 Comparison summary

The segmentation of the expert-labeled datasets demonstrates the achieved performance of the synthetic-only approach wrt. the performance of networks as trained directly on manually annotated images. The performance of the synthetic-only network is comparatively lower than the real-data training, however productive accuracy is achieved for identifying marine-growth (seaweed) and seawater, especially when applied to the SUIM dataset. A comparison of the per-class accuracy and IoU against the original dataset proposed algorithms are shown in Table 6.

**TABLE 6:** Dataset benchmark performance comparison, ‘-’ denotes not available in cited work.

Model	Ref.	Base architecture (backbone)	Global. acc	Mean. IoU	Acc. marine-growth	IoU marine-growth
*LIACi-DeepLabV3*	Waszak et al. (2023)	DeepLabV3	77.34%	75.28%	55.64%	52.20%
*LIACi-SegNet*	Waszak et al. (2023)	SegNet (ResNet50)	82.79%	80.63%	67.78%	63.62%
*LIACi (subset) w. ours*		DeepLabV3+ (Xception)	40.00%	22.49%	74.11%	37.75%
*SUIM-DeepLabV3*	Islam et al. (2020)	DeepLabV3	81.27 ± 2.30%	79.10 ± 2.34%	—	—
*SUIM-Net-VGG*	Islam et al. (2020)	VGG-16	86.97 ± 2.30%	84.14 ± 1.15%	—	—
*SUIM-Net-VGG*	Islam et al. (2020)	VGG-16	86.97 ± 2.30%	84.14 ± 1.15%	—	—
*SUIM-DatUS* ^*2*^	Kumar et al. (2024)	ViT-B8	64.67%	28.48%	—	—
*SUIM-STEGO*	Kumar et al. (2024)	ViT-S8	53.24%	24.76%	—	—
*SUIM-AIM++*	Vorndran and Roeck (2024)	AIM	67.8%	48.5%	—	—
*SUIM (subset) w. ours*		DeepLabV3+ (Xception)	66.13%	29.28%	75.70%	55.86%

### 3.3 Test results - Offshore video

**FIGURE 13:**
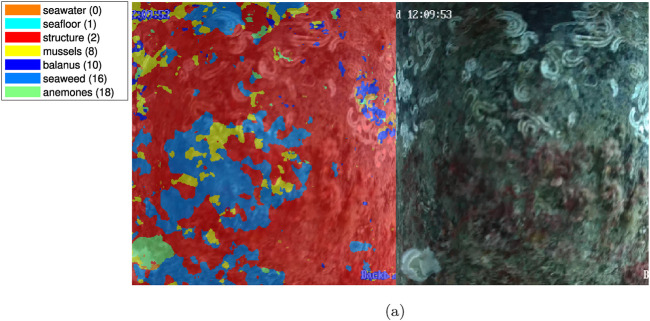
Example segmentations of offshore videos, courtesy of SubC Partner. **(A)** Offshore example 1.

An instance of the segmentation results for the offshore environment is shown on Figure 13. Concerning segmentation performance, the following can be noted: the sea anemones on the central segments of the image are segmented with reasonable accuracy, but those at the edge, where resolution is lower, are erroneously labeled as seaweed; the seaweed in the lower left-hand corner is labeled correctly but with some overlap on the anemones adjacent to it.

### 3.4 Test results - Coastal videos

**FIGURE 14:**
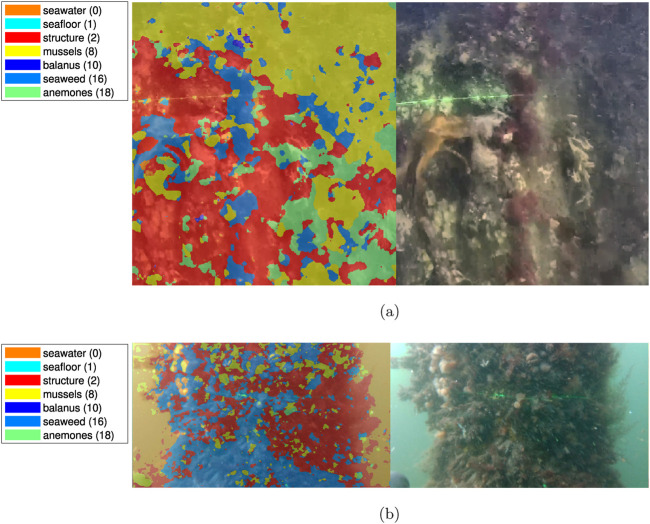
Example segmentation of coastal videos, courtesy of SubC Partner. **(A)** Coastal example, Fredericia Habor, courtesy of SubC Partner. **(B)** Coastal example, Aabenraa Habor, courtesy of SubC Partner.

A sample of the segmentation results for the coastal environment is shown on Figure 14. Concerning segmentation performance, the following can be noted: the seaweed in the central left section and the vertical centerline in the image have been labeled correctly. In the lower right quadrant, misclassification as anemones occurs, though no anemones are present in the test location. A considerable part of the shaded area towards the right and top of the image is incorrectly labeled as mussels for indeterminate reasons.

### 3.5 Test results - Ship hulls

**FIGURE 15:**
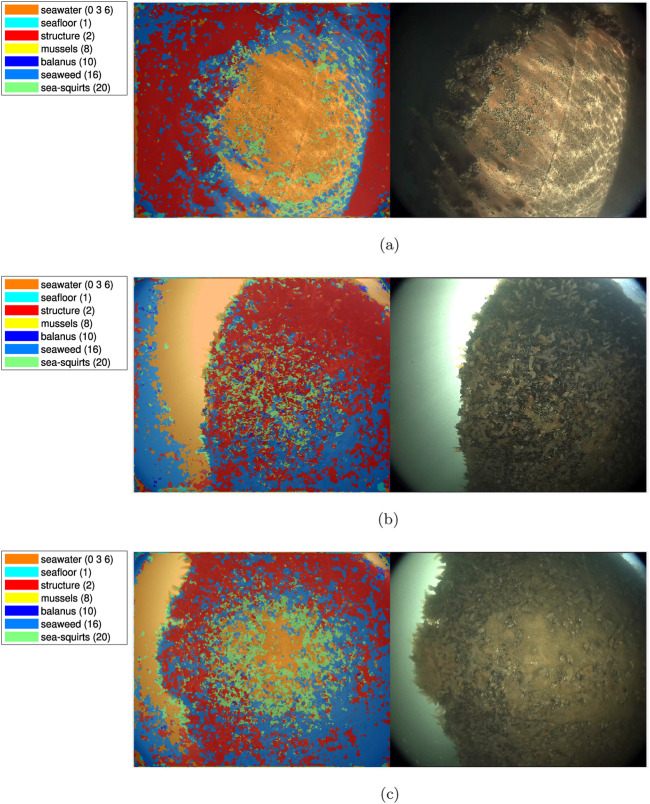
Example segmentation of ship hull videos, courtesy of Blue Atlas Robotics. **(A)** Ship hull example 1. **(B)** Ship hull example 2. **(C)** Ship hull example 3.

Examples from ship hull scenarios are shown on Figure 15. The out-of-bag performance for the ship hull videos is inferior to the structural and coastal videos. Chief sources of the reduced classification performance, relative to the offshore case are manyfold. The lower fidelity and compression artifacts of the input videos, are caused by increased compression of the raw images, and motion artifacts, are not captured in the synthetic data used for training. The presence of reduced color saturation is caused by the camera’s white balance and the presence of caustics due to sunlight from the surface also causes misclassification as evident in Figure 15A, in the right part of the image. In the image of Figure 15B, a significant part of the sea squirts in the center of the image is correctly classified, however, the surrounding mussels are incorrectly classified as structural surfaces. The performance in segmenting the boundary of the free water volume is illustrated on the left part of Figure 15C, where the upper part and central parts are captured well, including the details around the central sea squirts. However, issues remain in classifying where the color of the water is less distinct, such as in the lower left part of Figure 15C and the lower left corner of Figure 15B. These factors are not present to the same extent in the offshore test cases due to higher raw camera resolution and larger operational depths (reducing caustics).

## 4 Discussion

This study has focused on qualitatively evaluating semantic segmentation applied to underwater surfaces using neural networks. The neural networks have been implemented using a two-stage training approach consisting of transfer learning on real-world data for classification layers and purely synthetic data for the segmentation layers. The use of synthetic data is motivated by the high cost of acquiring and manually labeling real-world underwater data. The approach has been illustrated in the application of marine-growth segmentation, motivated by the extensive and varied presence of marine fouling on man-made structures.

The results demonstrate the utility of the synthetic dataset approach in training a usable neural network, with apparent usable results on real-world imagery. However, some areas of performance degradation remain, particularly in lower-resolution sectors of the images or where there is shade or caustics. A substantial false classification rate exists between the seafloor and seawater, which is, however, less relevant in the task of distinguishing between marine growth and structure; similarly, the increased false classification rate between Balanus and mussels is of less consequence for distinguishing between hard and soft marine-growth. Indeed, the misclassification between the marine growth species and the structural surface is more critical and should be addressed further in future work. This is particularly the case for mussels, which suffer from false positives and false negatives in several application scenarios. This could be due to the varying presentation of microfouling on the mussel’s exterior surfaces. A more thorough and strict definition of the classes would be beneficial for the marine-growth segmentation; for example, the difference between thin layers of marine fouling and thick layers consisting of multiple cohabiting organisms should be addressed, which is not captured in the presented approach, and is expected to have a substantial influential the he misclassification between almost clean but highly textured structural surfaces biofilm surfaces, macrofouling and more clearly delineated fouling species such as mussels and anemones.

As a general observation, the promising results achieved for offshore structures do not extend fully to ship hull applications, where the current misclassification remains unacceptably high. This discrepancy highlights the clear current limitations of the proposed segmentation approach across different applications. Improvement can be pursued through the development of more realistic and varied synthetic environments and by enhancing the fidelity of the sensor data. Introducing layered cohabitating species appears to be the most obvious enhancement to synthetic environments. For the sensor data, efforts should focus on improving the quality of optical information, including better color representation, reduced compression artifacts, and minimized motion blur. The present work has investigated the application of optical information for segmentation; however, incorporating additional sensor modalities, such as acoustic technologies, can enhance environmental information. Acoustic data are robust against optical losses and can potentially provide an additional depth dimension that is useful for distinguishing between different species. A prior study has already initiated the generation of synthetic acoustic data (Woock and Beyerer, 2017; Reitmann et al., 2021).

Importantly, validating the actual accuracy in the real-world dataset requires using of a ground-truth dataset. Due to the varied presentation of the marine growth, an expert-labeled dataset is most readily usable, however the available datasets often do not overlap completely with the synthetic data in terms of class delineation, which makes a direct comparison impossible, and necessitates the use of more encompassing superclasses which are less demonstrative of the achievable species performance.

Following the validation of the trained networks, they can be applied to marine-growth segmentation tasks in various industrial contexts, such as for the recurrent or continuous monitoring of marine-fouling load on structural components, through the implementation of edge-computing hardware aboard ROV’s and AUV’s, or through offline usage in historical data analysis. Furthermore, the approach can be expanded to other contexts by modifying the underlying virtual environment and generating additional data, such as seafloor piping or other types of subsea installations, or for analyzing natural habitats.

A natural avenue of further exploration is to evaluate and compare additional state-of-art neural network architectures with training on the synthetic data, especially given the rapid evolution of network architectures in both the underwater and other robotics domains (Ni et al., 2023; Juana Valeria Hurtado and Abhinav Valada, 2024; Xu et al., 2023); relatedly, a systematic exploration of additional modifications of the neural network for the concrete marine-growth segementation scenario could also be explored. For application in a real-world scenario, the developed network must be deployed for inference either as offline processing of captured video and images as presented in this work or as an online edge-processing solution. Generally, semantic segmentation in real time on an edge processing device remains computationally intensive, thus yielding low frame rates as elucidated in Lambert et al. (2023), where framerates of <3.5 frames per second are generally achieved on a modern edge-processing device (NVIDIA Jetson Nano 4 GB). However efficient network such as ESPNet achieves significantly better performance at ≈ 18FPS, and the expected continued development of edge-computation devices will most probably continue to improve the achievable segmentation performance. A similar adaptation is shown in Islam et al. (2020) where adaptations of the neural network provide an FPS performance gain.

## Data Availability

The datasets presented in this article are not readily available because the synthetic image dataset is commercial; however validation data is available on request. Requests to access the datasets should be directed to chrimai@energy.aau.dk.

## Footnotes

1 In the LIACi dataset, the background water is not directly labeled, so background class is assumed for all otherwise unlabeled pixels, similar to the synthetic renders.

2 In the SUIM dataset, the water surface and free water body is labeled together.
